# Diffusion-weighted imaging and FDG PET/CT: predicting the prognoses with apparent diffusion coefficient values and maximum standardized uptake values in patients with invasive ductal carcinoma

**DOI:** 10.1186/1477-7819-10-126

**Published:** 2012-06-28

**Authors:** Bo Bae Choi, Sung Hun Kim, Bong Joo Kang, Ji Hye Lee, Byung Joo Song, Seung Hee Jeong, Hyeon Woo Yim

**Affiliations:** 1 Department of Radiology, Chungnam University Hospital, Chungnam, Korea; 2 Department of Radiology, The Catholic University of Korea, Seoul, Korea; 3 Department of General Surgery, The Catholic University of Korea, Seoul, Korea; 4 Department of Preventive Medicine, Clinical Research Coordinating Center, College of Medicine, The Catholic University of Korea, Seoul, Korea

**Keywords:** Invasive ductal carcinoma, breast, magnetic resonance imaging, diffusion magnetic resonance imaging, positron emission tomography

## Abstract

**Background:**

FDG PET/CT and DWI are both functional modalities that indirectly represent the biological characteristics of cancer, but there are few studies exploring the association between the two modalities and prognostic factors. Our study attempted to evaluate the mutual association by comparing the prognostic factors, SUVmax value of PET/CT, and ADC values associated with diffusion imaging in invasive ductal carcinoma (IDC) patients.

**Methods:**

Patients with pathologically confirmed IDC were recruited. There were 118 patients who underwent MRI, including DWI, FDG PET/CT, and immunohistochemical staining of the surgical specimen. Histologic analysis was done on tumor size, lymph node metastasis, expression of estrogen receptors (ER), progesterone receptors (PR), human epidermal growth factor receptor 2 (HER2), Ki-67, and epidermal growth factor receptors (EGFR). The relationship among ADC values, SUVmax and prognostic factors were evaluated.

**Results:**

There was significant association between the ADC value and ER-positive and HER2-negative expression. Significant associations were noted between SUVmax and tumor size, lymph node metastasis, histologic grade, ER and PR expression, EGFR and Ki-67. However, there was no significant correlation between the ADC value and SUVmax.

**Conclusions:**

Even though there was no correlation between ADC and SUVmax, both indexes are useful for predicting the prognosis of IDC.

## Background

Breast cancer is a heterogeneous tumor that exhibits various patterns of progression, results and treatment responses. The management plans for breast cancer are determined in accordance with the preoperative tumor-nodes-metastasis (TNM) stage, histologic classification of postoperative TNM stage, and the levels of hormone receptor and molecular markers in the specimens [1]. Predicting the prognosis of breast cancer is very important for determining the direction of treatment; the conventional prognostic factors include tumor nuclear grade, tumor size, and the presence of lymph node metastasis. The immunohistochemical prognostic factors include hormone receptors such as estrogen receptor (ER), progesterone receptor (PR), human epidermal growth factor receptor 2 (HER2), and Ki-67. The role of 18F-fluorodeoxygluxose positron emission tomography/computed tomography (FDG PET/CT) reflects the glucose metabolism in accordance with the increase in cancer cell glycolysis, and it is used for the diagnosis of lesions, staging, recurrence, and treatment response [1]. FDG uptake aids in predicting the prognosis for primary breast cancer and is associated with several histopathological and immunohistochemical prognostic factors [2,3]. The diffusion-weighted image (DWI) is a modality used to evaluate the microstructural characteristics of water diffusion in biological tissues [4]. The apparent diffusion coefficient (ADC) is calculated by DWI. Malignant tumors with highly cellular lesions display low ADC values due to the inverse relationship with tumor cellularity [5,6]. Therefore, ADC is useful for distinguishing malignant and benign tumors, and DWI is useful for evaluating the tumor’s response to treatment. However, few studies on the association between ADC values and prognostic factors have been published, and some researchers say that there is no association [5,6]. FDG PET/CT and DWI are both functional modalities that indirectly represent the biological characteristics of cancer, but there are few studies exploring the association between the two modalities and prognostic factors [1]. Our study attempted to evaluate the mutual association by comparing the prognostic factors, maximum standardized uptake value (SUVmax) value of PET/CT, and ADC values associated with diffusion imaging.

## Methods

### Patients

From the pathology database, among the patients who had been confirmed with IDC through biopsies and surgeries from June 2008 to January 2010, the names of patients who had received DWI and PET/CT and undergone surgeries were collected. From these patients, ones who had received preoperative chemotherapies or adjuvant chemotherapies; ones who, although they had been diagnosed with breast cancer through ultrasound or mammography, had not shown lesions in magnetic resonance imaging (MRI) or PET/CT; and ones who had recurrences during postoperative follow-ups were all excluded. From that total of 142 patients, and excluding the 24 patients who showed non-mass in their MRI scans, 118 patients were included in this study.

### Imaging acquisition

The MRI images were acquired with a 1.5 T scanner (Achieva; Philips Medical Systems, Best, The Netherlands) and a 3.0 T scanner (Magnetom Verio; Siemens Medical Solutions, Erlangen, Germany) equipped with a breast coil. The MRI images with the Achieva scanner were performed using the following sequences: sagittal, fat-suppressed, fast spin-echo T2-weighted imaging sequence; axial DWI with a single-shot echo-planar imaging (EPI) with b factors of 0 and 1000 second/mm2; pre- and dynamic axial T1-weighted three-dimensional, fat-suppressed, fast spoiled gradient-echo sequence.

The MRI images from the Verio scanner were acquired using the following sequences: axial, turbo spin-echo T2-weighted imaging sequence; axial DWI with echo-planar imaging (EPI) with b factors of 0 and 750 seconds/mm2; pre- and post-contrast, axial T1-weighted fast low-angle shot (FLASH) three-dimensional, volume interpolated breath-hold examination (VIBE) sequence.

PET/CT studies were acquired on combined PET/CT in-line systems, either Biograph Duo or Biograph Truepoint (Siemens Medical Solutions, Knoxville, TN, USA). The acquisition time was two to three minutes per bed position. All patients were in supine position during PET/CT scanning. CT began at the orbitomeatal line and progressed to the proximal thigh (130 kVp, 80 mAs, and 5 mm slice thickness; 120 kVp, 50mAs, and 5 mm slice thickness). A PET scan followed immediately over the same body region. The CT data were used for attenuation correction, and images were reconstructed using a standard ordered-subset expectation maximization (OSEM) algorithm. The axial spatial resolution was 6.5 mm or 4.5 mm at the center of the field of view, respectively.

### Image analysis

DWIs were obtained along each of the x-, y- and z-axes. The ADC value was calculated according to the formula: $\text{ADC}=\left[1/\left(\text{b}2-\text{b}1\right)\right]\text{ln}\left(\text{S}2/\text{S}1\right)$, where S1 and S2 are the signal intensities in the regions of interest (ROI) obtained by two gradient factors, b2 and b1 (b1 = 0 and b2 = 1000 s/mm2 for the 1.5 T scanner; b1 = 0 and b2 = 750 s/mm2 for the 3.0 T scanner). For the measurement of the ADC value, one radiologist with four years’ experience of breast MRI manually placed a region of interest (ROI) that was slightly smaller than the solid portion of the tumor to ensure that cystic, necrotic portions of normal parenchyma were not included [1,3,5]. The mean ADC values were obtained.

One radiologist with six years’ experience of breast MRI reviewed the PET/CT report paper and SUVmax of the breast cancer was used.

### Histologic analysis

Pathologic reports were reviewed to determine the size, lymph node metastasis, and histologic grade. Immunohistochemistry was used to test for the expression of the following molecular markers: estrogen receptor (ER), progesterone receptor (PR), human epidermal growth factor receptor 2 (HER2), Ki-67, and epidermal growth factor receptor (EGFR). ER and PR positivity was defined as the presence of 10% or more positively stained nuclei in ten high-power fields. The intensity of c-erbB-2 staining was scored as 0, 1+, 2+ or 3+. Tumors with 2+ or 3+ scores were classified as HER2 positive, and tumors with 0 or 1+ were negative. EGFR was considered positive if membrane staining was observed. Ki-67 of > =15% was considered positive expression.

### Statistical analysis

Data are presented as the median and range for continuous variables and frequency with percentage for categorical variables.

To examine whether the ADC value and SUVmax value can provide prognostic information, the difference in ADC values and SUVmax values of each prognostic group was analyzed. In cases in which the prognostic groups were classified as the positive group and the negative group, the Mann–Whitney U test and Kruskal-Wallis for variables with non-normal distribution were used. To evaluate the correlation between ADC value and SUVmax, Spearman’s correlation coefficient was used. The statistical analyses above were performed with SAS software, version 9.1 (SAS Institute Inc. Cary, NC, USA). A two-tailed *P* value of <0.05 was considered statistically significant.

Written informed consent was obtained from the patient for publication of this report and any accompanying images.

## Results

The mean age of 118 patients was 52 years (range, 29 to 81 years). The mean value of tumor size, ADC mean and SUVmax was 27.5 ± 21.7 mm, 994.3 ± 217.0X10-6 mm2/s and 4.4 ± 3.8 (mean ± SD), respectively.

Histologic analysis was invasive ductal carcinoma (IDC) in 32 patients (27%) and IDC with ductal carcinoma *in situ* (DCIS) in 86 patients (73%). Histologic grades consisted of 33% (39/118) well differentiated, 50% (59/118) moderate, and 17% (20/118) poorly differentiated. In the immunohistochemical study, ER and PR positives were 89 patients (76%) and 74 patients (63%), respectively; HER2 positives were 50 patients (43%); EGFR were 23 patients (20%). Ki-67 was 25% on average.

**Figure 1:**
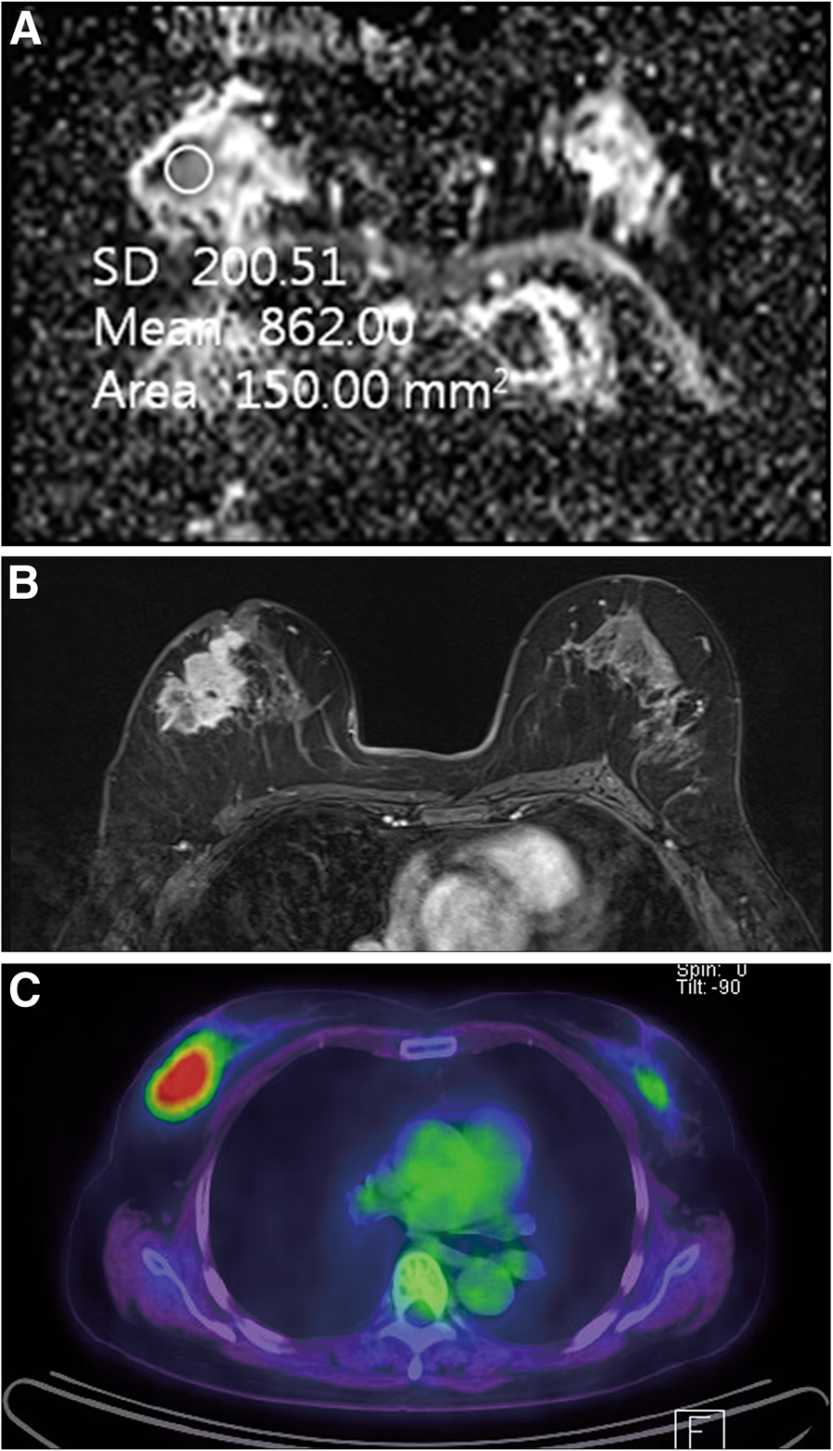
**A 65-year-old woman with invasive ductal carcinoma of the right breast.** On immunohistochemical study, ER and PR were negative. (**A**) ADC map shows ROI for measuring the mean ADC value. The calculated ADC value was 862 × 10^-6^ mm2/s, which was lower than mean ADC value (994 × 10^-6^ mm2/s). (**B**) Axial enhanced T1-weighted image after two minutes of contrast injection demonstrates a spiculated enhancing mass in the right breast. (**C**) FDG PET/CT image shows FDG uptake in the right breast with measured SUVmax as 7.3. ADC, apparent diffusion coefficient; ER, estrogen receptor: FDG PET/CT, 18 F-fluorodeoxygluxose positron emission tomography/computed tomography; PR, progesterone receptor, ROI, region of interest

**Figure 2:**
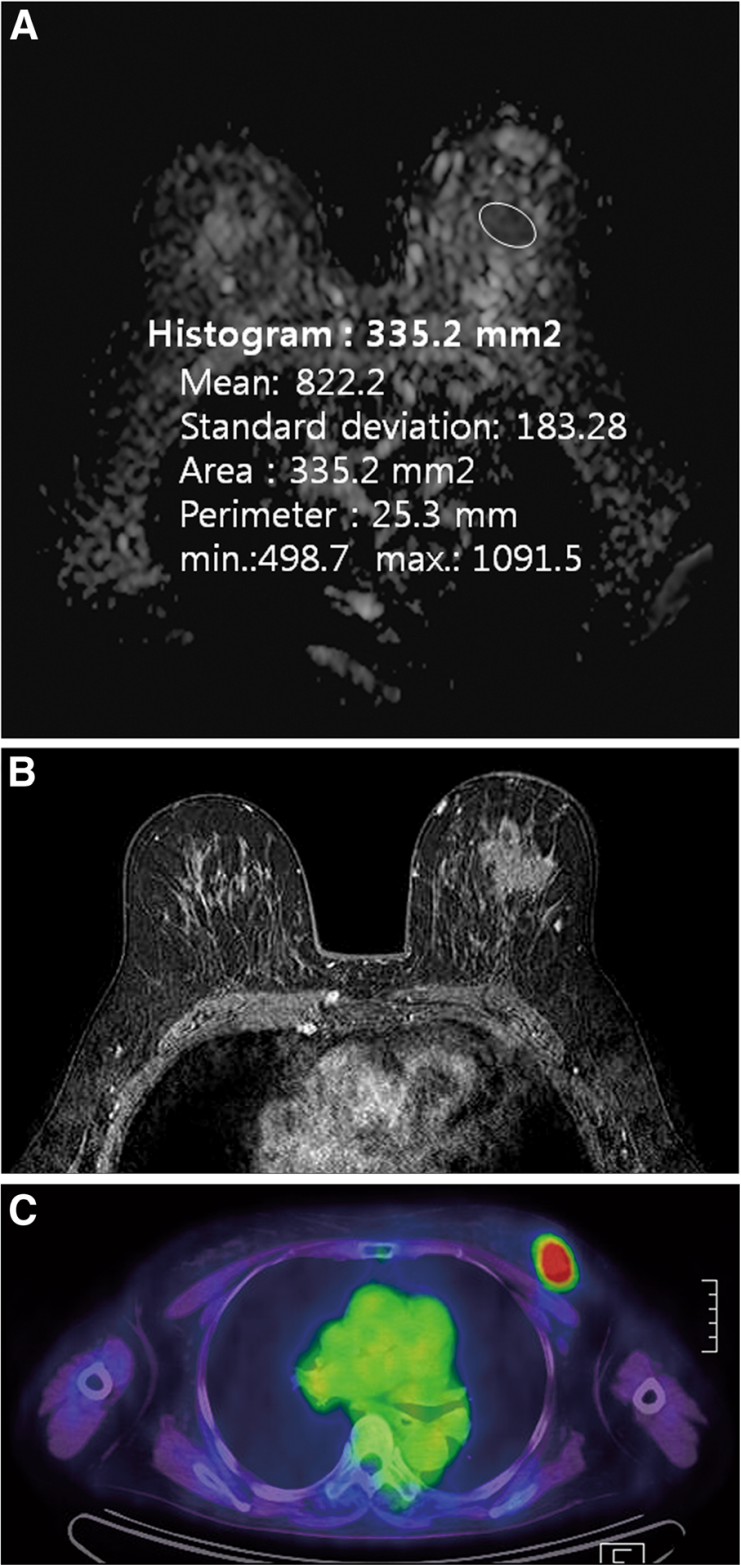
**A 65-year-old woman with poorly differentiated invasive ductal carcinoma of the left breast**. (**A**) On ADC map, calculated ADC value by ROI was 822 × 10^-6^ mm2/s, which was lower than mean ADC value (994 × 10^-6^ mm2/s). (**B**) Axial enhanced T1-weighted image demonstrates an irregular spiculated mass in the left breast. (**C**) FDG PET/CT image shows FDG uptake in the left breast. Measured SUVmax of breast cancer is 7.3. On histologic examination, axillary LN metastasis was noted in one LN. The results of immunohistochemical study were ER (−), PR (−), HER2 (−), EGFR (+) and Ki-67 70%. ADC, apparent diffusion coefficient; EGFR, epidermal growth factor receptor; ER, estrogen receptor: FDG PET/CT, 18 F-fluorodeoxygluxose positron emission tomography/computed tomography; HER2, human epidermal growth factor receptor 2; LN, lymph node; PR, progesterone receptor; SUVmax, maximum standardized uptake value

In the statistical analysis, the ADC mean showed statistically significant low values in the ER-positive expression group and HER2-negative expression group (*P* <0.05) (Table 1, Figure 1). The high SUVmax was shown to have correlations with the large tumor sizes, lymph node (LN) metastasis positives, high histologic grades, ER-negative expression, PR-negative expression, EGFR-positive expression, and high Ki-67 (Table 1, Figure 2). There was no correlation between the ADC mean and SUVmax (*P* = 0.786). The correlation coefficient r was −0.025.

**Table 1:** Associations of SUVmax and ADC with clinicopathologic prognostic factors in invasive ductal carcinoma

**Factor**	**Number of cases (n = 118)**	**ADC mean (X 10–6 mm2/s)**	***P* value**	**SUVmax**	***P* value**
Tumor size (mm)					
<20	49(41.88)	942.3(412.3-1793.7)	0.185	1.9(0–8.9)	<0001
20-50	57(48.72)	969.3(599.8-1555.5)		4.7(0–20.5)	
>50	11(9.40)	1061.2(661.4-1402.9)		5.1(1.2-15.2)	
LN metastasis					
negative	76(65.0)	970.2(412.3-1793.7)	0.655	2.5(0–18.7)	0.003
positive	41(35.0)	962.1(599.8-1555.5)		4.8(1.2-20.5)	
Histologic grade					
1	39(33.3)	962.9(412.3-1793.7)	0.832	2.1(0–10.5)	<0001
2	58(49.57)	966.7(565.2-1555.5)		2.9(0–18.7)	
3	20(17.1)	955.3(666.5-1402.9)		7.1(2.6-20.5)	
ER					
negative	28(23.9)	1043.6(764.1-1402.9)	0.009	5.3(0–18.7)	0.003
positive	89(76.1)	946.5(412.3-1793.7)		2.8(0–20.5)	
PR					
negative	43(36.8)	1012.99(753.5-1405.5)	0.055	4.6(0–20.5)	0.035
positive	74(63.3)	947.6(412.3-1793.7)		2.8(0–14.1)	
HER 2					
negative	67(57.26)	922.3(412.3-1793.7)	0.016	2.9(0–16.1)	0.529
positive	50(42.74)	1019.8(599.8-1555.5)		3.7(0–20.5)	
EGFR					
negative	92(80.0)	962.3(412.3-1793.7)	0.321	2.7(0–20.5)	0.001
positive	23(20.0)	1041.0(666.5-1402.9)		5.3(1.3-18.7)	
Ki-67					
<15	50(42.7)	962.7(412.3-1793.7)	0.998	2.3(0–8.6)	0.001
≥15	67(57.3)	969.3(568.4-1402.9)		4.8(0–20.5)	
Age					
<50	59(50.4)	971.1(412.3-1555.5)	0.577	3.2(0–16.1)	0.301
≥50	58(49.6)	959.6(565.2-1793.7)		3.0(0–20.5)	

* ADC, apparent diffusion coefficient; EGFR, epidermal growth factor receptor; ER, estrogen receptor: HER2, human epidermal growth factor receptor 2; LN, lymph node; PR, progesterone receptor; SUVmax, maximum standardized uptake value.

## Discussion

Both diffusing MR imaging and FDG PET/CT can provide important functional information for IDC with high cellularity. Diffusion MR imaging can provide information about the random water motion in tissues, and have a potential roles in the characterization of malignancy, including determination of lesion aggressiveness and monitoring response to therapy [5,7-10]. PET/CT visualizes the metabolic activities of tissue and provides information about the rate of glucose metabolism [2]. FDG PET/CT can detect enhanced glycolysis of breast cancer cells and can be used for diagnosing, staging, detecting recurrence, and assessing response to therapy [1,2]. Both diffusion MR imaging and FDG PET/CT may be related to tumor cellularity. Areas of high cellularity have more structures and cell membranes resulting in impedance of water motion, and have increased cellular proliferation. Therefore, ADC values are low and FDG uptake gets higher in high cellular tumors [11]. In contrast, areas with low cellularity are less resistant to water diffusion resulting in higher ADC value; scarce proliferative activity resulting in lower FDG uptake [11].

### Correlation between ADC value and prognostic factors

We studied the correlation between mean ADC value and prognostic factors such as tumor size, LN metastasis, histologic grade, age, and expression levels of ER, PR, HER2, EGFR, and Ki-67. Significant associations were noted between mean ADC and positive ER status (*P* = 0.009) and negative HER2 status (*P* = 0.016), which is similar to the results reported by Jeh *et al*. [6]. The ADC value is useful for the differentiation of malignant masses from benign masses or normal breast tissue, as malignant tumors have lower ADC values compared to benign masses [7,12-15]. Hatakenaka *et al*. [8] reported that the ADC value differed significantly between invasive and non-invasive ductal cancer. ER expression is associated with a favorable prognosis [16-18]. Tumors that display positive ER and PR expression respond well to adjuvant and palliative hormone therapy [4]. Ludovini *et al*. [19] explained that the reason for the lower ADC values observed in the ER-positive group as compared to the ER-negative group is that ER blocks the angiogenic pathway and reduces perfusion, which in turn affects the ADC value. Notably, HER2 is similar in structure to the human epidermal growth factor receptor (EGFR). When overexpressed, it inhibits the control mechanisms in normal cells, accelerates cell growth and division and can cause cancer. HER2-positive cells that are more likely to exhibit a malignant phenotype, accompanied by cell proliferation, invasion and metastasis [4,6]. In addition to cell growth, HER2 induces angiogenesis; thus, blood flow increases in tumors that are HER2 positive and hormone receptor negative [16]. Reduced perfusion in the HER2-negative group may explain the observed reduction in ADC values. Kim *et al*. [4] reported that ADC values were higher in tumors with increased HER2 expression, although there was no significant correlation between HER2 expression and ADC values.

There was no correlation between mean ADC value and histologic grade as determined by Ki-67 expression. Notably, one of the important indices of histologic tumor grade is tumor cellularity [6,14]. Ki-67 is a marker of tumor cellularity and is a benign nuclear antigen found in proliferative-phase cells [6,14]. Tumor grade and Ki-67 expression are also related to other proliferative diseases and recurrences [17], and cancer of lower cellularity is associated with improved prognosis [20]. Diffusion is difficult in areas of dense tumor cellularity because of the effective motion of water molecules [13]. There are some reports of a negative correlation between ADC values and tumor cellularity [7,8,15,21]. Woodhams *et al*. [21] reported that DWI is a unique marker of tumor cellularity. However, Yoshikawa *et al*. [22] and Kim *et al*. [4] reported that ADC value and tumor cellularity are not correlated. Previous reports found no correlation between Ki-67 expression and ADC value [6].

ADC mean was not correlated with LN metastasis. Breast cancer with ipsilateral axillary lymph node metastasis is the most important predictor of long-term survival [23,24]. In the case of LN metastasis, the prognosis is poor, with the prognosis worsening as the number of LN metastases increases [7]. The presence of LN metastasis is most important in conjunction with the expression of hormone receptors in the decision to proceed with conservative therapy [7]. Some authors have reported a correlation between LN metastasis and ADC value; the ADC value can help in avoiding unnecessary surgical staging of axillary LN [1,7]. However, Kim *et al*. [4] found no association between ADC and LN metastasis, which is similar to our results.

ADC values and tumor size were not correlated (*P* = 0.185). Tumor size is a very important factor in the future prognosis of breast cancer [7]. As tumor size increases, the likelihood of metastasis increases, and the overall survival rate decreases [7]. Although Kim *et al*. [4] and Nakajo *et al*. [1] stated that ADC and tumor size are not correlated, Kuroki-Suzuki *et al*. [9] stated that there is a significant correlation among ADC, pT1 and pT2-4. Razek *et al*. [7] also reported a correlation between tumor size and ADC. Based on a hypothesis that small lesions of less than 1 cm are difficult to detect in DWI, Razek *et al*. excluded lesions less than 1 cm in size. Because lesions less than 1 cm in size were not excluded in this study, which resulted in a difference in mean tumor size, comparison with the results obtained by Razek *et al*. [7] is difficult (in this study, the mean was 2.8 ± 2.2 cm; in the study by Razek *et al*., the mean was 3.9 ± 2.0 cm).

### Correlation between SUVmax and prognostic factor

SUVmax were associated with numerous prognostic factors such as tumor size, LN metastasis, histologic grade, and expression levels of ER, PR, EGFR, and Ki-67 among others; these findings are similar to those reported by previous studies [1-3,25]. Oshida *et al*. [2] used differential absorption ratio (DAR) as the FDG uptake index; the high DAR group demonstrated significantly worse prognoses with respect to both overall and relapse-free survival. Ueda *et al*. [25] stated that a high SUVmax is associated with higher relapse and mortality rates.

### Correlation between ADC mean and SUVmax

Mean ADC and SUVmax were not correlated (*P* = 0.786). There were reports of ADC values decreasing due to the fact that water diffusion becomes difficult in regions of high tumor cellularity. Furthermore, ADC and tumor cellularity are inversely proportional [7,8,15,21]. Several authors have reported that FDG uptake and cellularity are positively correlated [26,27]. Because SUVmax and ADC were expected to be inversely proportional to each other, the existence of any correlation between the results obtained with both modalities is currently being investigated further. Nakajo *et al*. [1] reported that the SUV values and ADC values were inversely proportional in the context of malignancy. Ho *et al*. [5] stated in their study of primary cervical cancer that SUVmax and ADC mean were not correlated. Our study used a larger sample size than that used by Nakajo *et al*. [1] and Ho *et al*. [5]. In contrast to the study performed by Nakajo *et al*. [1], our study did not separate the prognostic group into a ‘better’ group and a ‘worse’ group. In contrast to Nakajo *et al*. [1], who included only DCIS, our study only used IDC as subjects. Because the study by Ho *et al*. [5] investigated cervical cancer, the subjects differed from those included in the present study. The accurate identification of a clinical correlation between DWI and PET/CT requires further evaluations involving a variety of subjects. For now, the two modalities cannot be considered as interchangeable.

This study has some limitations. First, because there were many cases in which ADC values were difficult to measure for non-mass lesions, 24 lesions were excluded due to inaccuracy of the ROI measurement. Due to the variation in histologic values, this exclusion of lesions may have introduced variability to the study. Second, although detecting lesions less than 1 cm was considered difficult with DWI, this study included all such small lesions; however, ACD values were not measured in these small lesions. Third, there was no correlation established between the histologic prognostic factors and the actual clinical prognosis follow-up. Further evaluations that complement these limitations are necessary.

## Conclusion

The ADC values were associated with several prognostic factors of IDC (tumor size, Ki-67, age, and so on.). The SUVmax was associated with multiple prognostic factors including the tumor size, LN metastasis, histologic grade, ER, PR, EGFR, Ki-67, and so on. DWI and FDG PET/CT are different image modalities representing different biologic aspects of cancer. Even though there was no association between the ADC values and SUVmax in IDC, these two modalities might play a complementary role in detecting the prognosis of IDC.

## Competing interests

The authors have no competing interests to declare.

## Authors’ contributions

BBC and SHK participated in conception of the study, data collection and analysis, and drafted the manuscript. BJK, JHL, BJS, SHJ, and HWY participated in data collection and analysis. All authors read and approved the final manuscript.
